# Novel optical optode for selective detection and removal of ultra-trace level of mercury ions in different environmental real samples

**DOI:** 10.1038/s41598-024-76571-y

**Published:** 2024-11-02

**Authors:** Hager A. Dayra, Magdy Y. Abdelaal, Magdi E. Khalifa, A. B. Abdallah

**Affiliations:** 1 Chemistry Department, Faculty of Science, Mansoura University, Mansoura, 35516 Egypt; 2 Chemistry Department, Faculty of Science, New Mansoura University, New Mansoura City, Egypt

**Keywords:** Optical optode, Thiosemicarbazone, Digital color analysis, Spectrophotometry, Removal, Real samples, Environmental sciences, Chemistry

## Abstract

Owing to the high cost and unavailability of different analytical techniques, there is an urgent need to develop new techniques not only for detecting but also removing mercury ions in real samples. Thus, an optical chemical sensor based on the anchoring of phenanthraquinone monophenylthiosemicarbazone in a plasticized cellulose triacetate membrane was fabricated and applied to the recognition and removal of mercury ions from aqueous solutions. The synthesized optode was characterized by FT-IR, SEM, AFM, and thermal analysis. Several parameters, including the pH, temperature, contact time, washing solvent, and washing time, were optimized. Under optimal conditions, a promising optode film platform was utilized for sensing mercury ions, and the concentrations were calculated based on colorimetric analysis (Histogram, RGB) of digital images, visualization, and spectrophotometry. Also, an optical optode was used for complete adsorption of mercury ions from aqueous solutions. In addition, the regeneration of the synthesized optode was evaluated using 0.1 mol L^− 1^ nitric acid, which effectively removed all adsorbed mercury ions. The obtained data indicated good linearity in the sensing and adsorption of Hg^2+^ over a concentration range of 0.005–5000 µgL^**−** 1^ with a low limit of detection (LOD = 0.066 µgL^− 1^) and limit of quantification (LOQ, 0.22 µgL^**− 1**^). Furthermore, it showed good distinctions in the presence of coexisting ions, high stability (five months), good applicability, and reproducibility (RSD = 1.31%), making it a promising sensor for Hg^2+^ detection. On the other hand, the kinetic studies revealed that the pseudo-second-order was the best model for describing the adsorption behavior of mercury ions on the optode surface. Also, the thermodynamic parameters indicate spontaneous (ΔG^0^ < 0) and endothermic (ΔH^0^ < 0) reactions. Also, the maximum adsorption capacity was found to be 73.2 mg g^− 1^. Thus, the optodes were successfully applied for the detection and/or removal of Hg^2+^ in different real samples, including cucumber, fish, soil, and water samples, with excellent recoveries of 98.1–99.5%.

**Supplementary Information:**

The online version contains supplementary material available at 10.1038/s41598-024-76571-y.

## Introduction

Optical chemical sensors (OCS) or optodes have been considered as alternative artificial receptors for the rational design of highly specific complementary binding sites for certain ions and/or molecules using appropriate chelating agents^1–6^. The selectively incorporated ionophore in OCS is the key compound that captures the target ions from the bulk solution and forms a colored component. Owing to their tailor-made affinity and specificity for a target analyte, optical optodes are an effective approach that can be utilized not only to detect various metal ions, but also to completely adsorb/remove the target ions at the optode surface. For chemical sensing, the colorimetric signal is the cheapest and most widely used technique for chemical analysis. It is based on the measurement of the color intensity and its correlation to the concentration of a certain analyte. Optical signals can be easily detected using the naked eye and/or spectrophotometric equipment^7–10^. Furthermore, the concentration of each target species can be measured by analyzing the color recorded by optical sensors using color coordinates (red-green-blue (RGB), XYZ, L*a*b, and other systems)^11,12^. The chromaticity parameters, including color variation, absorbance, intensity of color, and reflectance of light, were calculated as functions of the absorption or reflection spectra using the Adobe Photoshop CS6 (64Bit) program on a personal laptop. Digital images of the OCS were captured using a smart electronic camera and/or scanner and then inserted into the histogram tool to obtain a linear concentration range of the measured metal ions. However, the regular spreading of a specific active agent on the optode surface enables the analytes to interact equally with all adsorbent particles, which achieves a high adsorption capacity per adsorbent amount. Therefore, OCS have attracted considerable interest for sensing and removing different heavy metals, particularly mercury ions, owing to their high chemical stability, mechanical properties, large specific surface area, and strong adsorption power.

Mercury is considered to be one of the most hazardous heavy metals because of its accumulative and persistent nature in the environment and biota. Organic mercury (Methylmercury (Me-Hg)) is produced by naturally occurring biomethylation processes in marine environments by microorganisms. Me-Hg eventually finds its way into aquatic creatures’ diets and into human bodies when fish are consumed. Mercury binds to proteins or thiols that contain sulfhydryl (-SH) groups through sulfur present in the amino acids cystine, cysteine, and methionine, as well as the cysteine-derivative taurine, which is abundant in bile. Since all the receptors found in and on cell membranes are composed of proteins, different types of mercury can attack these receptors. Higher eukaryotes have a large number of protein transport systems that contain cysteine. Because these systems have cysteine residues exposed to the extracellular environment, they may interact with mercury. The range of availability of a protein molecule for regular metabolic activity is blocked or diminished when mercury attaches to one of the amino acid residues in receptor proteins. When Hg binds to one of the amino acid residues in receptor proteins, it blocks or attenuates the range of available protein molecules for normal metabolic function. Thus, highly negative effects on human health are mainly caused by damage to brain function, the nervous system, and kidneys, ataxia, muscle weakness, numb limbs, difficulty speaking, as well as skin rashes^13–15^. However, its existence has been reported in numerous fields, including agricultural and industrial products such as plastic, paper, paint, and pharmaceutical products. Thus, mercury ions can easily enter the food chain and biomagnify, starting with plankton and herbivorous fish and ascending to carnivorous fish and sea mammals. According to the WHO and FAO recommendations, the maximum permissible limits of inorganic mercury ions for the human body have been established as > 10 nM for drinking water and > 50 nM for food^14,15^. Therefore, it is crucial to detect trace amounts of Hg^2+^ with a high sensitivity and selectivity. Inductively coupled plasma atomic emission spectrometry (ICP-AES), inductively coupled plasma optical emission spectrometry (ICP-OES), and high-performance liquid chromatography (HPLC) have been widely utilized to detect Hg^2+^^1,16–21^. However, these methods suffer from low sensitivity and selectivity, short linear range, are time-consuming, are available only in centralized laboratories, and require expensive and sophisticated equipment^22–26^. As expected, optodes have received considerable attention from researchers for the analysis of trace pollutants because of their high accessibility, simplicity, ease of preparation, low cost, and high sensitivity and selectivity. According to our literature survey, only a few reports have been published on Hg^2+^ detection using chemical optodes. However, a short linear range of detection, short stability, high response time, and low sensitivity and selectivity have been reported^1,3,11,12^.

Therefore, a sensitive and selective optical chemical optode was fabricated to detect and eliminate mercury ions from different real samples. To the best of our knowledge, this is the first study to detect and remove Hg^2+^ using the same chemical optode via the fast reversible interactions between PPT and Hg^2+^. After the transparent polymer film was prepared, full characterization was conducted using Fourier transform infrared spectroscopy (FT-IR), atomic force microscopy (AFM), scanning electron microscopy (SEM), and thermogravimetric analysis (TGA). Nitric acid, hydrochloric acid, sodium hydroxide, and ethylenediaminetetraacetic acid (EDTA) were used to leach the detected mercy ions. Furthermore, the chemical stability of the optode sensor for the sensing and removal of mercury ions was checked over two months. In addition, other analytical parameters, including the pH, contact time, temperature, and selectivity for Hg^2+^ detection, were optimized for the synthesized optodes. The kinetic and thermodynamic parameters of the adsorption process were determined. The linear range was determined using different color-measuring techniques (optical and/or spectrophotometry and/or optode analysis using histograms).

## Experimental

### Chemicals and materials

All chemicals and reagents used in this study were of analytical reagent grade and were used without further purification. Dimethylformamide (DMF, 99.8%), chloroform (99%), anhydrous sodium acetate (NaOAC, 99.9%), nitric acid (86%), and ethanol (EtOH, 99%) were purchased from Sigma-Aldrich (Steinheim, Germany). Glacial acetic acid (AcOH; 99%), tri-n-butyl phosphate (98%), hydrochloric acid (37%), and 2-nitrophenyl octyl ether (99%) were obtained from Fluka (Buchs, Switzerland). Cellulose triacetate (CTA) and 4-phenyl3-thiosemicarbazide (98%) were obtained from Alfa Aesar (Heysham, United Kingdom). It is worth mentioning that all glass vessels were immersed in nitric acid (0.1 mol L^− 1^) for 2 days and then washed with double distilled water (DDW, resistance: 18.2 MΩ at 25 ◦C), dried at 30 ◦C, and directly utilized for the experiments.

### Experimental details

#### Preparation of stock solution

Stock solutions of Hg^2+^ (5 × 10^− 2^ mol L^− 1^) were prepared by dissolving an appropriate amount (1.35 g) of mercuric chloride in 100 mL DDW and stored at 4 ◦C until use. Furthermore, 0.1 mol L^− l^ NaOAC (0.82 g in 100 mL) and AcOH (0.57 mL glacial AcOH in 100 mL DDW) were freshly prepared before use and mixed with different aliquots to obtain an acetate buffer solution (acidic buffer). While, 0.1 mol L^− l^ of each boric acid (0.61 g in 100 DDW), sodium tetraborate (3.81 g in 100 DDW), and sodium chloride (0.58 g in 100 DDW) were prepared and then mixed with different aliquots to obtain a basic borate buffer solution.

#### Preparation of the chelating agent

The chelating agent utilized in the optical chemical sensor should have high selectivity toward the target ions and form a colored compound. Therefore, phenanthraquinonemonophenylthiosemicarbazone (PPT) was selected as the most selective ligand. The synthesis of PPT was previously reported by Khalifa et al., starting with 4-phenyl3-thiosemicarbazide with a high yield of 86%^27^. Specifically, 0.334 g of 4-phenyl3-thiosemicarbazide (2 mmol) was dissolved in hot ethanol and then mixed with 0.416 g of 9,10-phenanthraquinone dissolved in the least amount of glacial acetic acid. The resulting solution was refluxed at 110 °C for 1 h. The resulting precipitate was filtered, washed several times with EtOH, and recrystallized from EtOH to obtain the desired PPT agent (melting point, 198 °C). Thin-layer chromatography was performed on aluminum sheets precoated with aluminum oxide (Merck, Darmstadt, Germany) to monitor PPT synthesis.

#### Membrane preparation

A transparent semisynthetic optode membrane was synthesized by inserting a chelating agent in cellulose triacetate (CTA) polymer in the presence of different plasticizers. Briefly, 50 mg of CTA was dissolved in 20 mL chloroform, followed by the addition of 150 mg of 2-nitrophenyl octyl ether and 100 mg of tri-n-butylphosphate with stirring for 30 min. Subsequently, PPT (25 mg) was dissolved in chloroform (10 mL) and added to the CTA solution with continuous stirring for 30 min. The solution was cast in a glass culture dish (flat bottom, 6 cm in diameter) and dried at room temperature for three days. Membranes 40–42 μm thick were cut into pieces suitable for use as sensors.

#### Assay procedure for mercury detection

The chemical interaction between mercury ions and the synthesized optical sensor at its surface was evaluated by inserting a fixed optode in a suitable buffer solution containing an appropriate amount of Hg^2+^ (0.005–5000 µgL^**− 1**^) at various temperatures (25–60 ^o^C) for different time intervals. Moreover, for selectivity studies, a series of different concentrations of interfering ions, such as Cu^2+^, Mg^2+^, Ca^2+^, Zn^2+^, Cd^2+^, Na^+^, and Fe^2+^, were added to a specific amount of Hg^2+^ and shaken with the synthesized optode under optimum conditions. The loaded Hg^2+^ was removed by washing the optode with nitric acid (0.01 mol L^− 1^).

#### Batch adsorption procedure

All the sorption experiments were performed using batch techniques. Different concentrations of Hg^2+^ were stirred with a fixed dose of dried optode (0.05 g, 0.5 cm × 0.5 cm) at various pH ranges (2–8), variable temperatures (20–60 °C), and different time intervals. The mixtures were shaken in a thermostatic shaker bath at 35 °C until equilibrium was achieved. After that, the supernatant was filtered through a cellulose membrane (pore size = 0.45 μm), and the equilibrium concentrations of the metal ions were detected by ICP-OES. Furthermore, the concentration of adsorbed mercury metal ions was determined by spectrophotometric and/or color analysis of the sensing optode. The percentage of recovery of the heavy metal extract was calculated using the following equation:

$$\:\%\text{R}=\frac{(\text{C}\text{i}\:\--\:\text{C}\text{f})\:}{\text{C}\text{f}}\times\:100$$

where C_i_ and C_f_ are the initial and final concentrations of residual chemical ions (mg L^− 1^), respectively.

#### Application

**Fig. 1:**
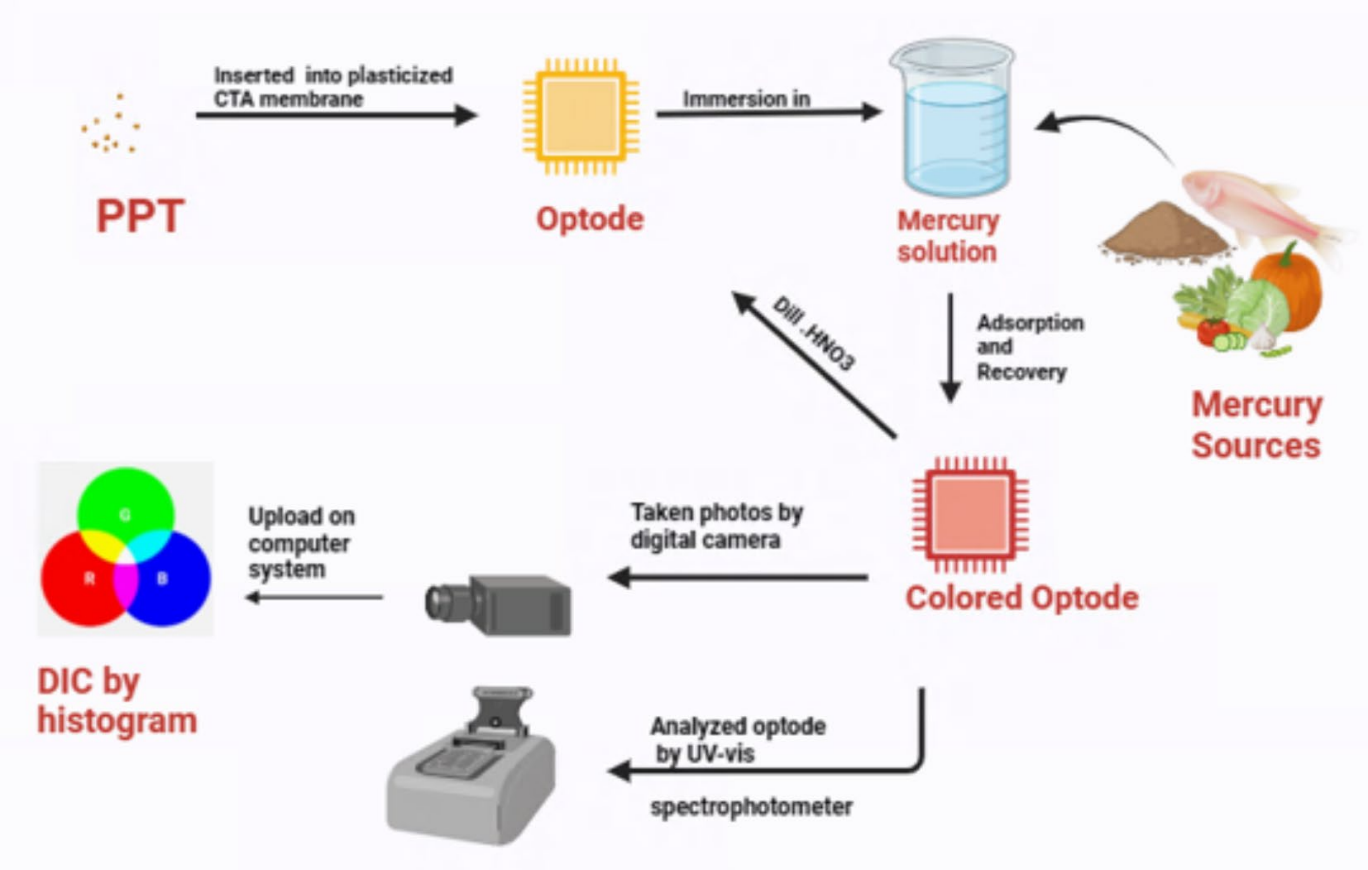
Detection and/or adsorption steps of mercury ions in real samples.

According to Fig. 1, mercury ions were detected and/or adsorbed from different real samples, including cucumber, fish, soil and water.

##### Vegetable sample

Cucumber samples were collected from the Metro Mall market (Mansoura, Egypt) and preserved in a plastic container for 30 min before reaching the laboratory. Subsequently, the samples were stored at (4 ± 1 °C) until further use. Before Hg^2+^ detection, each sample was washed multiple times with tap water, followed by double-distilled water, and then cut into small parts using a blender with microcutters. Subsequently, it was dried in an electric oven at 80 °C for 4 h. The crushed sample was dissolved in 100 mL distilled water and sonicated in a sonicator bath (frequency; 45 kHz, Temp; 30 °C) for 10 min until a homogeneous solution was obtained. Then, each synthesized sample was filtered through a cellulose membrane (pore size = 0.45 μm) and then buffered with borate solution.

##### Fish sample

The fish samples were purchased from a local mall. The samples were transferred to an experimental laboratory in a glass container containing ice at 0 °C. The backbones of the fish were then removed using a sharp knife. Subsequently, it was crushed in a blender using a microcutter. Subsequently, in a measuring flask, the synthesized sample was mixed with 4 mL of conc. HNO_3_-H_2_O_2_ (2:1v/v) and stirred for 15 min at room temperature. The mixture was then heated to near dryness, and another amount of the acidic mixture was added until the reddish-brown vapors stopped. The obtained sample was buffered with borate and diluted with 50 mL DDW. Finally, Hg^2+^ was detected and adsorbed according to the proposed procedure, with respect to its calibration curve.

##### Soil sample

10–20 g of dried soil sample was inserted into a100 mL micro-kjeldahl flask and digested in the presence of excess hydrogen peroxide. The sample was filtered through Whatman No. 40 filter paper into a 25- mL flask and neutralized with dilute ammonia. The solution was then diluted with deionized water to the desired concentration. Useful aliquots (1–2 mL) were transferred to volumetric flask and buffered with a borate buffer solution. Finally, Hg^2+^ was adsorbed and detected using the proposed procedure with respect to its calibration curve.

##### Water samples

Tap, Nile, and lake water samples were collected from various locations in Egypt. Each sample was filtered through a cellulose membrane (0.45 µµm) to get rid of any suspended materials. After that, the pH of the sample was adjusted to 2.0 and preserved in a dark polyethylene bottle (kept in the refrigerator) until Hg^2+^ detection.

### Characterization techniques

The chemical composition of the synthesized polymers was investigated using a Fourier-transform infrared spectrometer (FT-IR; Thermo Scientific, NICOLETiS10, USA). Atomic force microscopy (AFM; Shimadzu, Wet-SPM9600, Japan) and scanning electron microscopy (SEM; JEOL JSM.6510LV, Japan) were used to examine the roughness and porosity of the polymer films. The stability and chemical decomposition of the polymers were evaluated by thermogravimetric analysis (TGA) in nitrogen atmosphere at a heating rate of 20 °C min^− 1^. The concentration of hydrogen ions was adjusted during the chemical process by using a digital pH meter (HI 8519; Hanna Instruments, Italy). An Agilent 5100 inductively coupled plasma-optical emission spectrometer (ICP-OES; Agilent Technologies. Melbourne, Australia) was employed to detect the mercury metal content to assess the accuracy and validity of the proposed optical sensor. In addition, Ultrasonic Bath (Fisher Scientific Ultrasonic Bath CPX8800) was utilized to form a homogeneous solution of different vegetable samples.

## Result and discussion

### Characterization

#### SEM and AFM

**Fig. 2:**
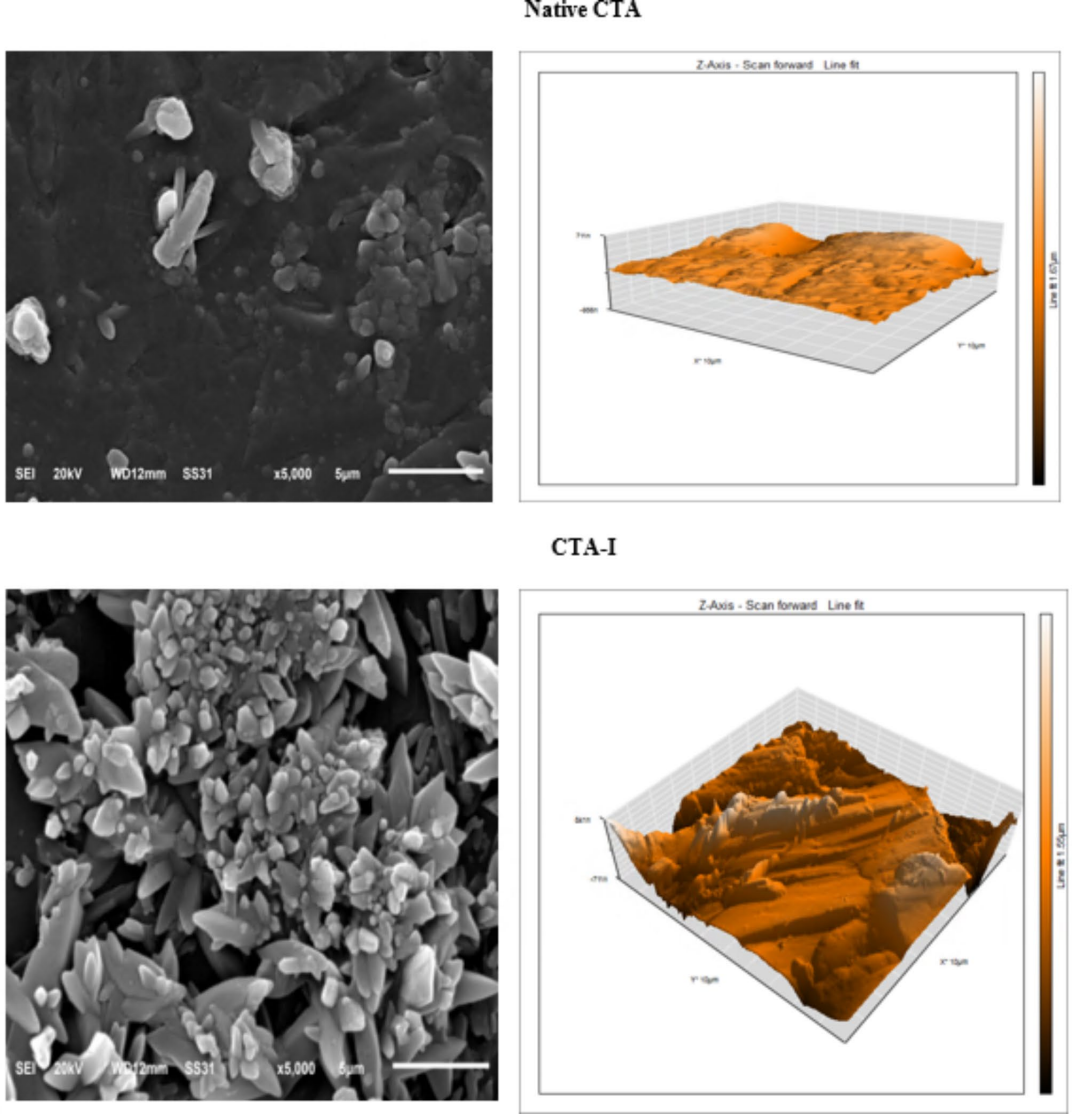
The SEM and AFM images of the native CTA, and CTA-I.

The surface morphologies and roughness of the native polymer surface (CTA) and the doped polymer surface (CTA-I) were elucidated using SEM and AFM. As shown in Fig. 2, a distinct variation is observed in the doped polymer surface after ionophore immobilization. The surface of the polymer was completely covered by thiosemicarbazide, and the roughness of the CTA-I polymer surface increased from 83.962 nm to 149.19 nm. Furthermore, the height of the polymer film was enhanced from 127.19 nm to 196.98 nm and indicating larger surface area.

#### FT-IR

The chemical structures of the native CTA and CTA-I were characterized using FT-IR spectroscopy. As shown in Fig. S1 (Supplementary Material), the absorption peaks at 3500, 1523, and 1596 cm^–1^ in the native CTA spectrum are assigned to O**–**H, C=C, and C= stretching, respectively^28^. Furthermore, the presence of nitro, phenyl, and phosphate groups at 1528, 1450, and 1150 cm^− 1^, respectively, emphasized the anchoring of the plasticizer on the polymeric surface. In addition, a sharp absorption peak at 1100 cm^− 1^ was observed in the CTA-I spectrum, confirming the successful insertion of chelating thiosemicarbazide into the synthesized optode.

#### Thermal analysis

Thermogravimetric analysis (TGA) was performed using a TGA-50 H thermogravimetric analyzer, and the samples were heated from room temperature to 650 °C at a rate of 20 °C/min under an inert nitrogen atmosphere. TGA thermographs of the optode films are shown in Fig. S2 (Supplementary Materials). The relative thermal stabilities of the optodes were assessed by comparing their weight losses in different temperature ranges. The first stage of weight loss 38.9 wt% of the total weight of optode film is occurred in temperatures 33.95–285.9 °C with loss of water and volatile organic matter, while the second stage 48.5 wt% at temperature 285.94–424.59 °C with losses of nitrogen, sulfur and oxygen. Finally, the remaining optode is decomposed by CTA at 424.59–610.94 °C.

### Preliminary investigation of the mercury complex formation

**Fig. 3:**
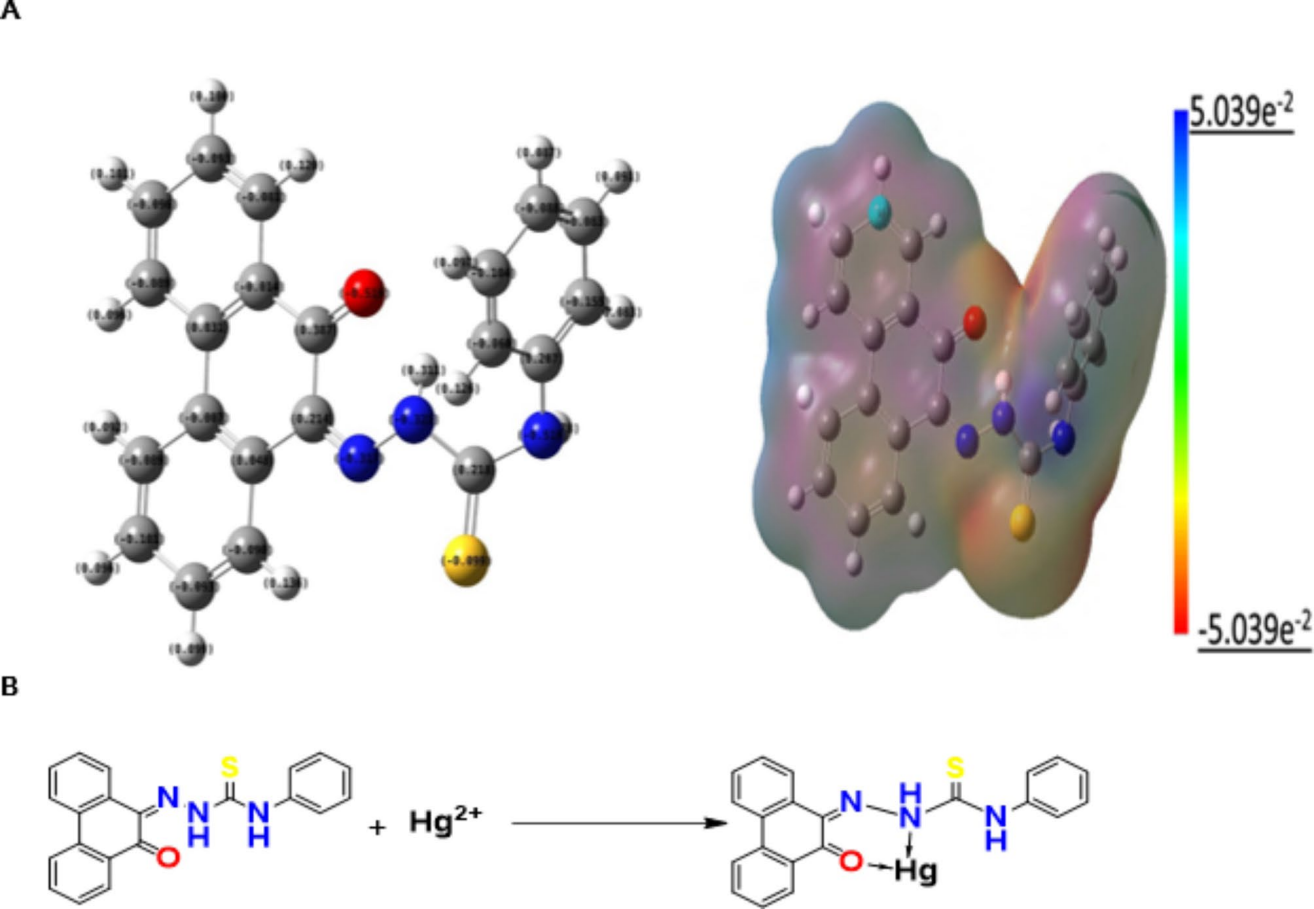
Molecular electrostatic potential map of the PPT agent (**A**), and the chemical interaction of the ppt, ligand with the mercury ions (**B**).

First, a molecular electrostatic potential (MEP) map was developed to predict the reactive sites in the PPT reagent, which easily captures mercury ions in the solution. As shown in Fig. 3A, oxygen and nitrogen (close to the C = S group) in PPT are the most donating sites with the highest electron densities of -0.519 eV and − 0.321 eV, respectively. Based on the obtained results, the chemical interactions between the proposed chelating agent and mercury ions are summarized in Fig. 3B.

### Parameters

#### pH

The efficiency of metal ion sensing and/or adsorption using an optical sensor is significantly influenced by the hydrogen ion concentration. Thus, the effect of pH was studied in the range of 2–9. As exhibited in Fig. S3 (Supplementary Material). Mercury sensing and/or adsorption sharply increased with decreasing concentrations of hydrogen ions, reaching their maximum sensing value at pH 8. Subsequently, the chemical sensing diminished slightly at higher pH values. The protonation of different functional groups of the PPT agent at lower pH values decreased its chemical binding mercury ions, while lowering the response at higher pH could be attributed to the formation of mercury hydroxide. It is worth mentioning that complete removal of Hg^2+^ ions in the concentration range of 0.005–5000 µgL^− 1^ was achieved at pH 8. Therefore, for further experiments, the borate buffer at pH 8 was selected to optimize the pH of solutions in order to obtain maximum sensitivity and reproducibility.

#### Effect of temperature

The chemical sensing and adsorption of mercury ions by the synthesized optode were investigated at temperatures ranging from 25 to 65 °C. As shown in Fig. S4 (Supplementary Material), the sensing and adsorption efficiencies were enhanced by increasing the temperature until they reached a maximum value at 35 °C. As the temperature increased, the efficiency decreased slightly and/or remained constant, which may be attributed to the possible damage of the active sites at the optode film.

surface causing lower adsorption capacity of mercury. All the mercury detection and adsorption experiments were performed at 35 °C for 20 s. In addition, thermodynamic parameters, including the standard entropy change (ΔS^o^), standard enthalpy change (ΔH^o^), and standard Gibbs free energy change (ΔG^o^), were estimated (Table S1(Supplementary Material)^29^. The obtained results indicated a spontaneous chemical interaction between the mercury ions and thiosemicarbazide derivative, PPT, on the surface of the synthesized optode. In addition, the positive values of enthalpy and entropy show that the proposed ions were adsorbed onto the interface through an endothermic process with increasing randomness.

#### Effect of ionophore concentration

Different modified optical sensors were prepared with different percentages of PPT ionophore (20–60%) for mercury ion adsorption and sensing. As presented in Fig. S5 (Supplementary Material), the optical sensing and adsorption recovery increase rapidly with increasing the percentage of PPT ionophore till reached to maximum value at 50% PPT. The lower sensing and removal efficiencies at low percentages of PPT could be due to the decreasing number of PPT molecules required to fix mercury ions at the optode film. Moreover, a sharp decrease in the chemical recovery and sensing at the 60% PPT-optode was observed, which may be attributed to the accumulation of PPT in a limited space giving a chance for intermolecular hydrogen bonding or steric hindrance probability, consequently decreasing the number of functional groups that reacted with the proposed ions. Thus, the chemical optode with 50% PPT was selected as the optimum sensor for mercury ion detection and the complete removal of Hg^2+^ from solution under optimum conditions.

#### Effect foreign ions

**Fig. 4:**
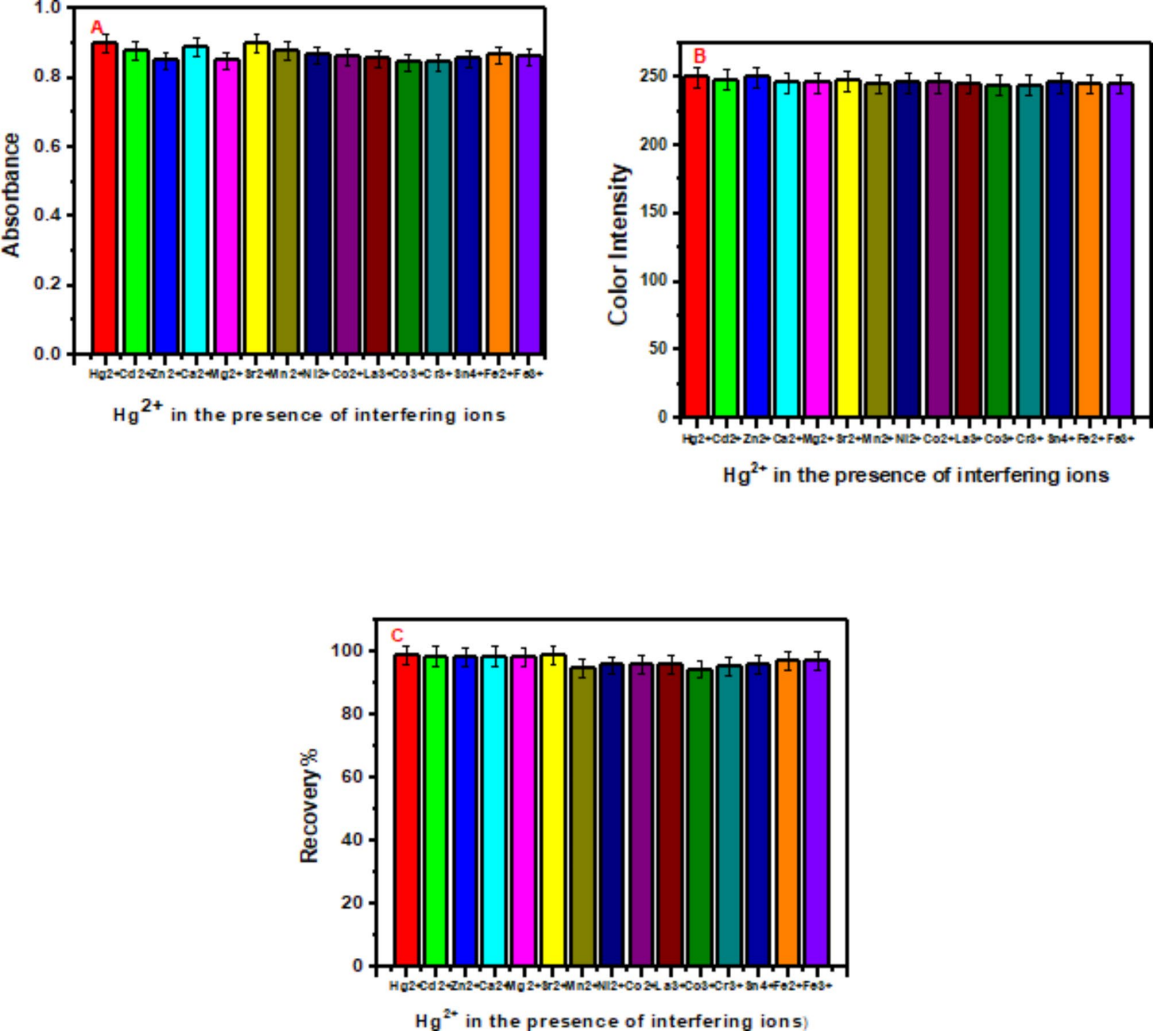
The absorbance signal of mercury ions in the presence of other interfering species (**A**), chemical analysis of optodes using histograms (**B**) and effect of different interfering ions on adsorption of mercury ions and recovery of mercury ions in the presence of different interfering ions (**C**). The sensing and adsorption of mercury ions was elaborated in the presence of Cd^2+^and Zn^2+^while there was 1 mL of 0.05 mol L^− 1^Na_2_S_2_O_3_, and 25 mL of 20% potassium cyanide solution.

Owing to the presence of mercury ions with high concentrations of coexisting ions, the selectivity of the proposed sensor should be evaluated. Therefore, the suitability and selectivity of the synthesized optode were examined by monitoring the mercury ions in the presence of different interfering ions, including Cd^2+^, Zn^2+^, Ca^2+^, Mg^2+^, Sr^2+^, Sn^4+^, Fe^2+^, Fe^3+^, Co^3+^, Cr^3+^, Zn^2+^, Mn^2+^, Ni^2+^, Co^2+^, and La^3+^. As shown in Fig. 4(A-C), high selectivity and sensitivity (in adsorption and/or sensing) of the optical optode were observed. Furthermore, there was no significant change in the chemical performance of the optode in the presence of 100-fold Ca^2+^, Mg^2+^, Sr^2+^, Mn^2+^, Ni^2+^, Co^2+^, Sn^4+^, Fe^2+^, Fe^3+^, Co^3+^, Cr^3+^, Zn^2+^, and La^3+^due to the specific interaction of PPT with mercury ions. Notably, the optode performance decreased by 81% of its original value in the presence of 100-fold Cd^2+^, Fe^2+^ and Zn^2+^. To overcome this drawback, 1 mL of 0.05 mol L^− 1^ Na_2_S_2_O_3_ was added to the proposed solution to mask the cadmium ions, 25 mL of 20% potassium cyanide solution was added to mask the zinc ions, and 5 mL of hydrogen peroxide was inserted to convert the Fe^2+^ to Fe^3+^.

#### Reusability of the optical optode

To achieve highly sensitive and reproducible sensors, the adsorbed mercury ions should be eluted from the optode surface. Regeneration of the synthesized optode was evaluated using different eluting solvents, including nitric acid, hydrochloric acid, EDTA, and NaOH. As expected, As expected, all these eluents could liberate mercury from the optode with different proportions, however in this work; the totally leaching of the detected mercury ions was accomplished using 0.1 mol L^− 1^ nitric acid. In addition, the chemical response of the optode enhanced accompanied by an increase in the leaching time (Fig. S6 (Supplementary Material)) until it reached a maximum value at 70 s. After 70 s, the chemical response remains constant. These results indicate that the complete removal of mercury ions was achieved after 70 s; therefore, this period was selected as the optimum leaching time required to regenerate sensing efficiency of the optode. However, after the recovery process, the complete removal of adsorbed mercury ions was achieved by 0.1 mol L^− 1^ nitric acid at 7 min (Fig. S6C (Supplementary Material)).

#### Stability of the optical optode

The chemical stability of the synthesized optical optodes was investigated over a long term (several months). The chemical sensor was inserted at an appropriate concentration of mercury ions for six months. Notably, a slight change in the optical density of the optode was observed after long-term storage (i.e.,>5 months) in a glass culture dish at room temperature. It is worth mentioning that the stability of this optode is higher than that sensors use physisorbing probe molecules. In contrast, the adsorption efficiency of the synthesized optode was affected (≤ 89%) after 3 months.

#### Adsorption capacity

To assess the chemical interactions of mercury ions with the optical optode, chemical adsorption and sensing were performed under isothermal conditions. The Langmuir (Eq. 1) and Freundlich (Eq. 2) isotherm equations were applied to evaluate the adsorption of ions on the optode surface^30^.

1 $$\:\text{C}_{\text{e}}/\text{q}_{\text{e}}=\text{C}_{\text{e}}/\text{q}_{\text{m}}+1/\text{K}_{\text{L}}\text{q}_{\text{m}}\:$$

2 $$\:\text{L}\text{n}\:\text{q}\text{e}=1/\text{n}\:\text{L}\text{n}\:\text{C}_{\text{e}}+\text{L}_{\text{n}}\text{K}_{\text{f}}\:$$

where C_e_ is the concentration of the metal ion in solution at equilibrium (mg/L), q_e_ is the amount of metal ion adsorbed on the optode at equilibrium (mg/g), q_m_ is the amount of metal ion adsorbed to form a monolayer coverage (mg/g), K_f_ and K_L_ is the Freundlich and Langmuir adsorption equilibrium constants, respectively. As exhibited in Fig. S7 and 8 (Supplementary Material), the adsorption of mercury ions process fits the Langmuir model to the Freundlich model (depending on the correlation coefficient), which emphasizes that the monolayer adsorption of target ions was accomplished at the optode surface. In addition, a high adsorption capacity (73.2 mg g^− 1^) was noticed for the prepared optode (Table S2 (Supplementary Material)).

#### Analysis of chemical optode for Hg^2+^detection

**Fig. 5:**
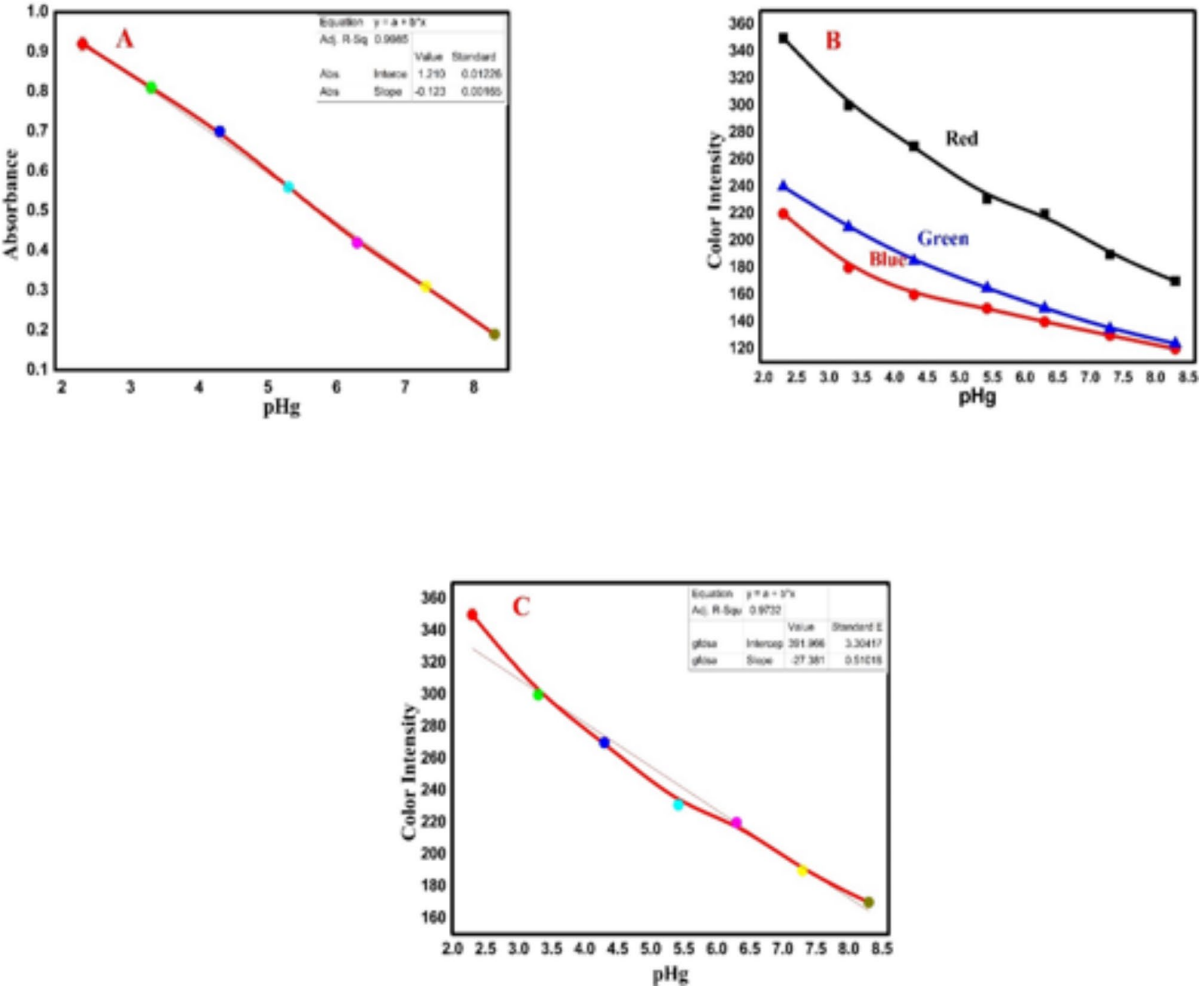
Linear correlation between the spectrophotometric absorbance (**A**) and RGB component absorbance (**B**) with the concentration of mercury ions. Relationship between the mercury ions concentrations and the mean integer value for each RGB component intensities (**C**).

Under optimum conditions, the colorimetric sensor was immersed in an appropriate concentration of mercury ions for 20s (optimum equilibration time), and then the solid chemosensor was collected by suction on filter paper (25 μm diameter). The oval region in the middle of the optical sensor sample was cropped using an Elliptical Marquee Tool. The amount of mercury was detected by the naked eye, Digital Image-based Colorimetric analysis, and UV–vis spectrometry. Chemical sensing of the optical chemosensor was performed via visual detection using an iPhone 14 Pro Max mobile camera (12 megapixels). As shown in Table 1, there is a wide difference in the color changes after the reaction of mercury ions with PPT, which is immobilized on the polymer surface. In addition, absorption spectra of the synthesized colored optodes were obtained, and the maximum absorption was recorded at 600 nm (Fig. 5A). Therefore, the analytical signals for the solid phase spectrophotometry were measured at 600 nm and then plotted against the mercury concentration. Furthermore, each colorimetric sensor was analyzed in terms of intensity in RGB coordinates (red, green, and blue) with the Adobe Photoshop “Histogram” tool on a personal laptop. All resulting data were listed in a spreadsheet (Microsoft Excel 2013) and plotted using Origin Pro 2016 (64 bit). As shown in Fig. 5B and C, the intensities of the R, G, and B channels depend on the concentration of mercury. To achieve the high accuracy and efficiency of the proposed technique, the intensity of the color, which has the highest slope (red color channel), was plotted against the concentration of mercury ions. Finally, the proposed method was validated by detecting Hg^2+^ in various real samples, including cucumber, fish, soil, and water.

**Table 1:**
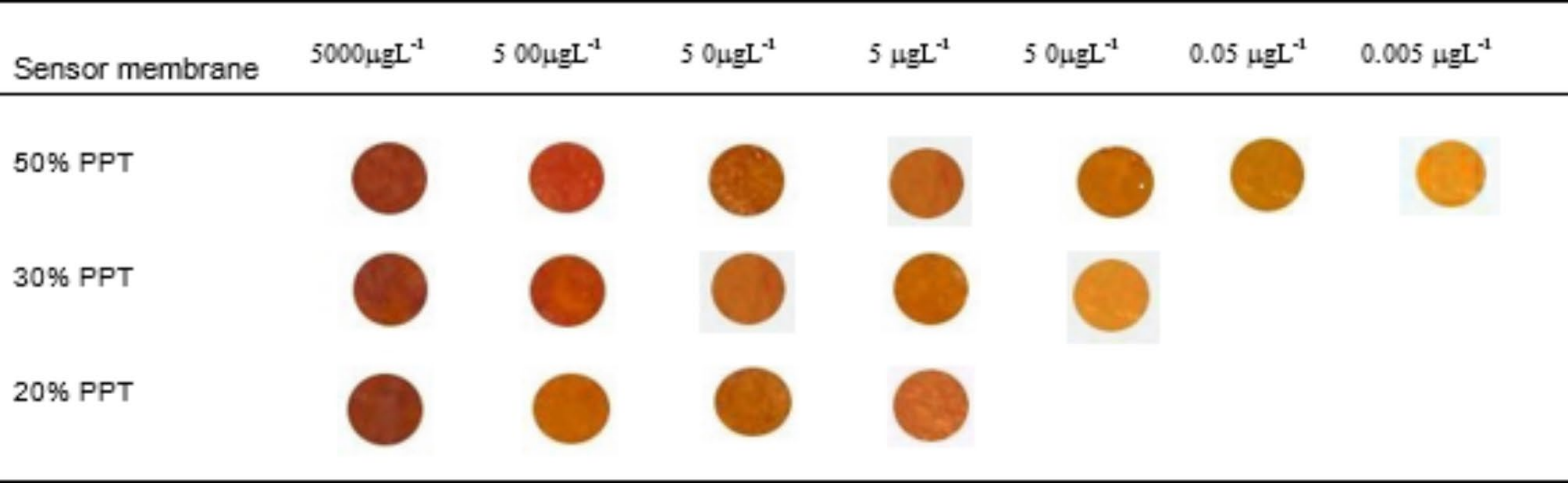
Performance of the single-indicator-based sensing platform toward mercury ions.

#### Analytical features

The high sensitivity and selectivity of the proposed optical film could be attributed to the presence of a PPT chelating agent as an ionophore that chemically interacts with the Hg^2+^ through nitrogen and oxygen atoms (Fig. 2). The determination of mercury ions in different samples was performed using colorimetric approaches, such as UV-visible analysis, histograms (color analysis of optode (Red, Green, and Blue), and visual methods. Under optimized conditions, the chemical sensing responses of the synthesized optodes were recorded using these techniques in the concentration range 0.005–5000 µgL^− 1^. Moreover, the chemical optode exhibited the complete adsorption of mercury ions within the same linear range. A typical linear chemical response of the optical optode was displayed, with a low limit of detection (LOD, 0.066 µgL^− 1^) and limit of quantification (LOQ, 0.22 µgL^− 1^). All chemical measurements were performed in triplicate. The chemical stability of the optical sensor membrane was examined by reacting it with mercury ions after storage of the membrane for five months in a glassy petri dish (for sensing and/or adsorption). Compared to other techniques, the proposed method exhibited long-term stability, good intermediate precision, and acceptable repeatability (Table S3(Supplementary Material)).

### Applications

To assess the analytical efficiency of the proposed method, an optical optode was used for Hg^2+^ detection in cucumber, fish, soil and water samples. In addition, the inter- and intra-day precision were evaluated by spiking each sample with different concentrations of Hg^2+^ (0,5.0, 10.0 µg L^− 1^). The determination of mercury ions was repeated four times, the mean value was elucidated, and the data are presented in Table S4 (Supplementary Material). As can be seen, the recoveries obtained were ranged from 98.1 to 99.5%, with low limit of relative standard deviations (RSD ≤ 1.31%). To verify the accuracy of this approach, the mercury ion concentration in each sample was measured using ICP-OES. The good agreement between the data obtained by our technique and those obtained by sophisticated ICP-OES emphasizes the applicability of the proposed technique for Hg^2+^ determination in various real samples with high accuracy and precision.

## Conclusion

A new, simple, sensitive, and selective approach for the detection of Hg ions was developed using synthesized optical chemical sensors based on Digital Image-Based Colorimetric Analysis and spectrophotometry. The optode, which was created using the PPT chelating agent, showed high sensitivity over a wide range (0.005–5000 µgL^**−** 1^**)** of distinguishable mercury metal analytes up to 0.066µgL^− 1^. Under optimized conditions, complexation between the PPT agent and Hg^2+^ is executed in a weakly basic medium at pH 8.0, which is a prerequisite for attaining high selectivity and complete recovery of Hg^2+^. As expected, the optode exhibited significant selectivity (sensing and/or removal) in the presence of other common coexisting ions (Ca^2+^, Cd^2+^, Zn^2+^, Mg^2+^, Sr^2+^, Mn^2+^, Ni^2+^, Co^2+^, Fe^2+^, Fe^3+^, Sn^4+^, Cr^3+^, Co^3+^, and La^3+^). In addition, it showed high stability (five months), environmental friendliness, and economically feasible production. It is worth mentioning that the synthesized optical optode was used for Hg^2+^ removal at the same concentration as the sensor, with high recovery. The synthesized optode was applied for the sensing and removal of mercury ions in fish, cucumbers, and natural water samples, with satisfactory results in terms of accuracy (98.1–99.5%) and precision (RSD ≤ 1.31%). These promising results open up a new approach for developing optical optodes for the detection and adsorption of various significant metal ions.

## Electronic supplementary material

Below is the link to the electronic supplementary material.

Supplementary Material 1

## Data Availability

All data generated or analysis during this study are available from the corresponding author upon reasonable request.
