# Supplementary material for: Novel optical optode for selective detection and removal of ultra-trace level of mercury ions in different environmental real samples
Source: Sci Rep. 2024 Nov 2;14:26400. doi: 10.1038/s41598-024-76571-y (PMC11531490; doi:10.1038/s41598-024-76571-y)
Supplement: Supplementary file 1 — Supplementary Material 1 [file 41598_2024_76571_MOESM1_ESM.docx]

**Novel optical optode for selective detection and removal of ultra-trace level of mercury ions in different environmental real samples**

Hager A. Dayra^a^, Magdy Y. Abdelaal^a^, Magdi E. Khalifa^a^, A. B. Abdallah^a,b^*

^a^ Chemistry Department, Faculty of Science, Mansoura University, Mansoura 35516, Egypt

^b^ Chemistry Department, Faculty of Science, New Mansoura University, New Mansoura

City, Egypt

^*^E-mail: [ahmed.bahgat@mans.edu.eg](mailto:ahmed.bahgat@mans.edu.eg)

**Supplementary Material**

**Figure caption**

**1-** Fig. S1; FT-IR of the CTA, and CTA-I optodes.

**2-** Fig. S2; Thermal analysis

**3-**Fig. S3; Effect of hydrogen ion concentrations on of mercury ion sensing on absorbance (A), chemical analysis of optodes using histograms (B) and adsorption of mercury ions and recovery (C).

4- Fig. S4; Effect of the temperature on mercury ion sensing on absorbance (A) , chemical analysis of optodes using histograms (B) and adsorption of mercury ions and recovery (C)

5- Fig. S5; Effect of the concentration of PPT in the optode surface on mercury ion sensingon absorbance (A), chemical analysis of optodes using histograms (B)and adsorption of mercury ions and recovery (C).

6-Fig. S6; Effect of the leaching time, for removing the mercury ions which adsorbed during the determination process, on absorbance (A), chemical analysis of optodes

using histograms (B) and adsorption of mercury ions and recovery (C).

7-Fig. S7; Freundlich isotherms for the adsorption of different concentration of Hg(II)

8-Fig. S8; The linear form of the Langmuir plots for the adsorption of Hg (II) onto the optode at room temperature

**Table caption**

1. Table S1. Thermodynamic parameters of the chemical interaction between the mercury ions with the synthesized optode.
2. Table S2. Langmuir and Freundlich constants.
3. Table S3. Comparison between our developed methods and other techniques for the detection of Mercury.

4-Table S4. Detection and Adsorpition of Hg (II) μgL**^-1^** from real samples using optode.

**Figures**


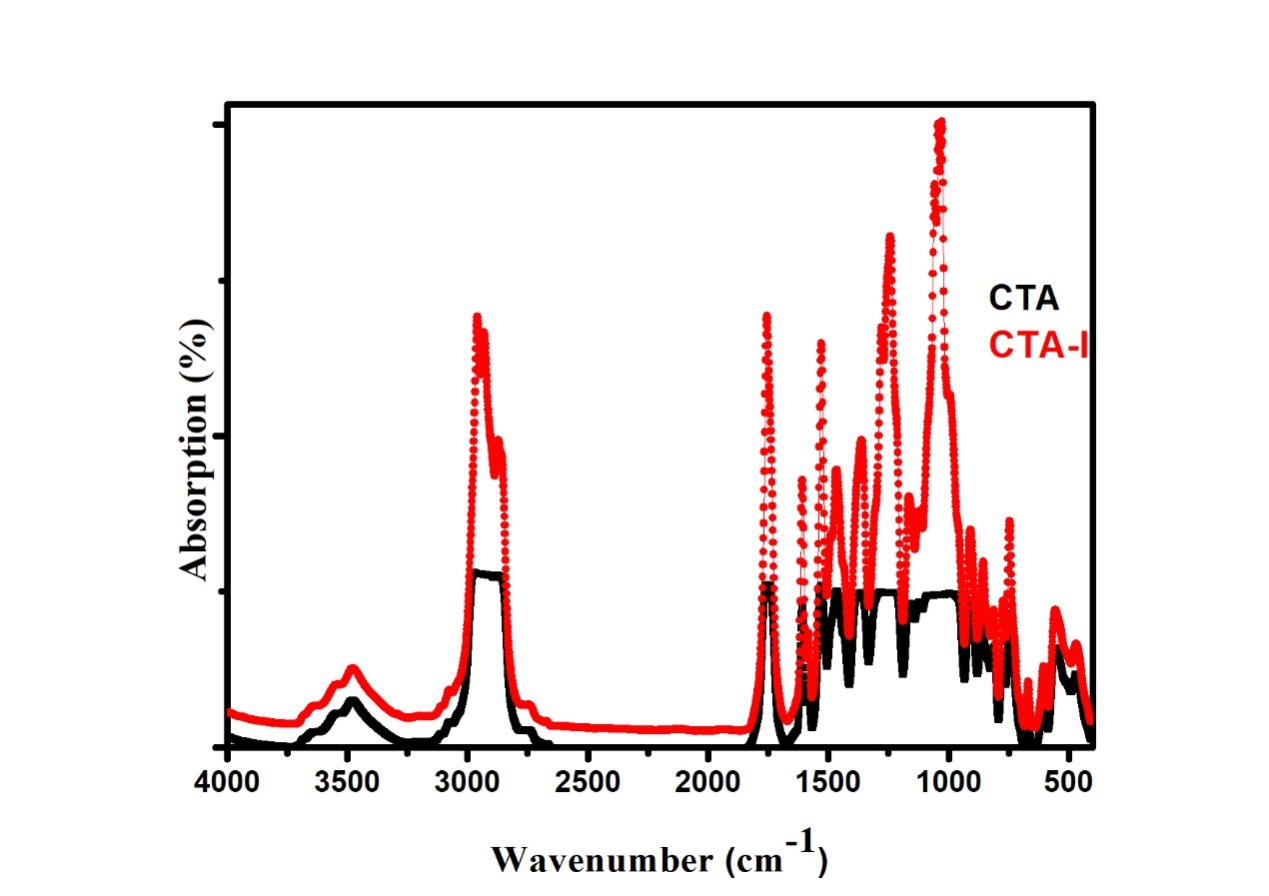


Figure. S1


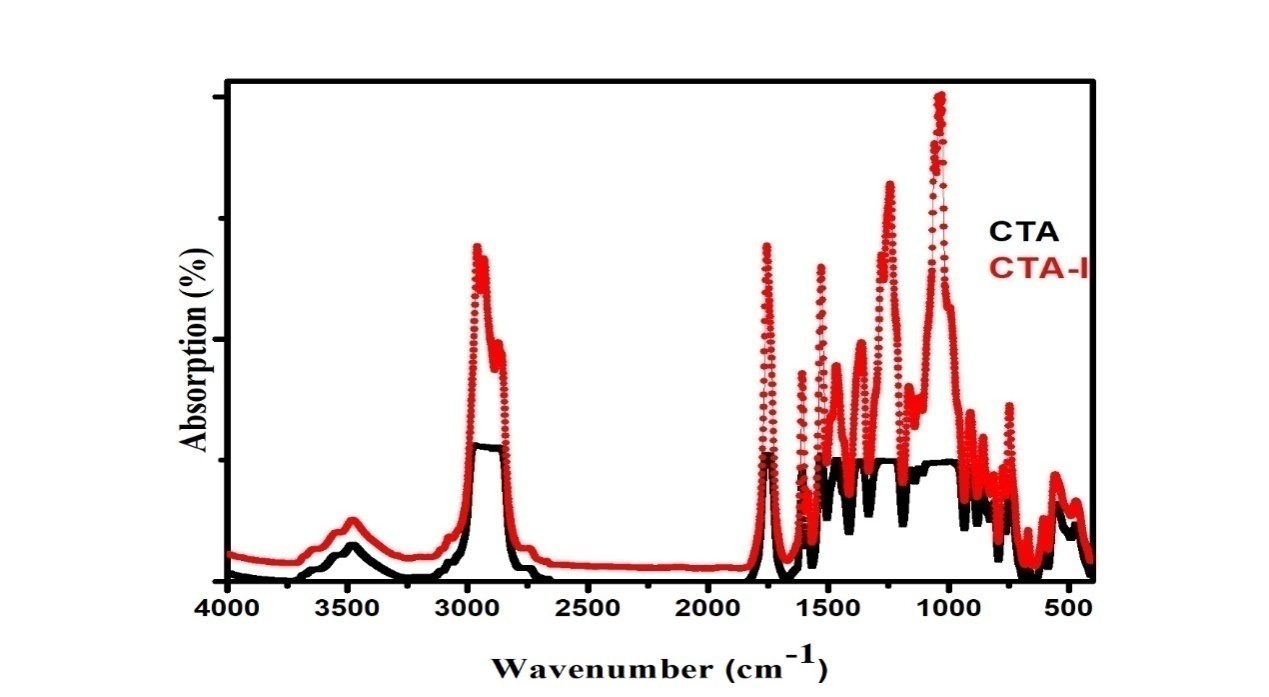

Fig. S2

| 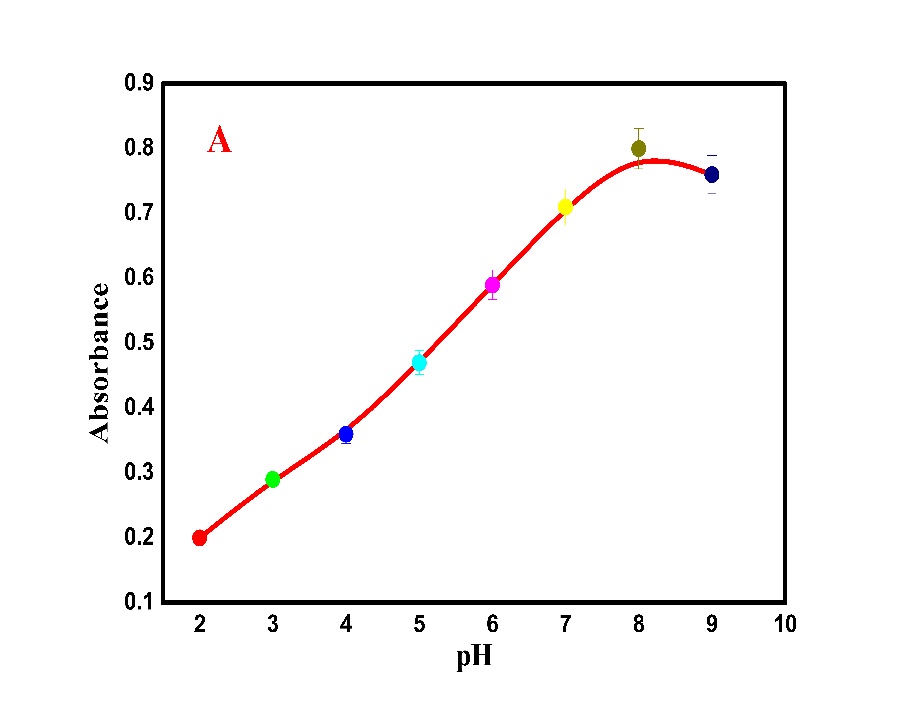 | | | 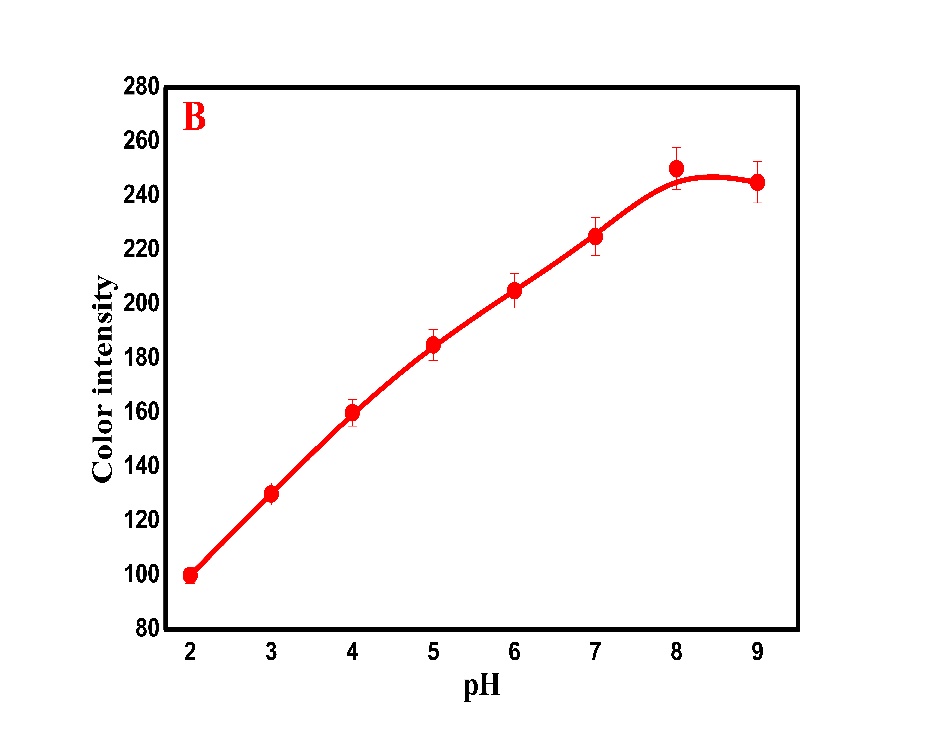 | | | | |
| --- | --- | --- | --- | --- | --- | --- | --- |
| Fig. S3. | | | | | | | |
| 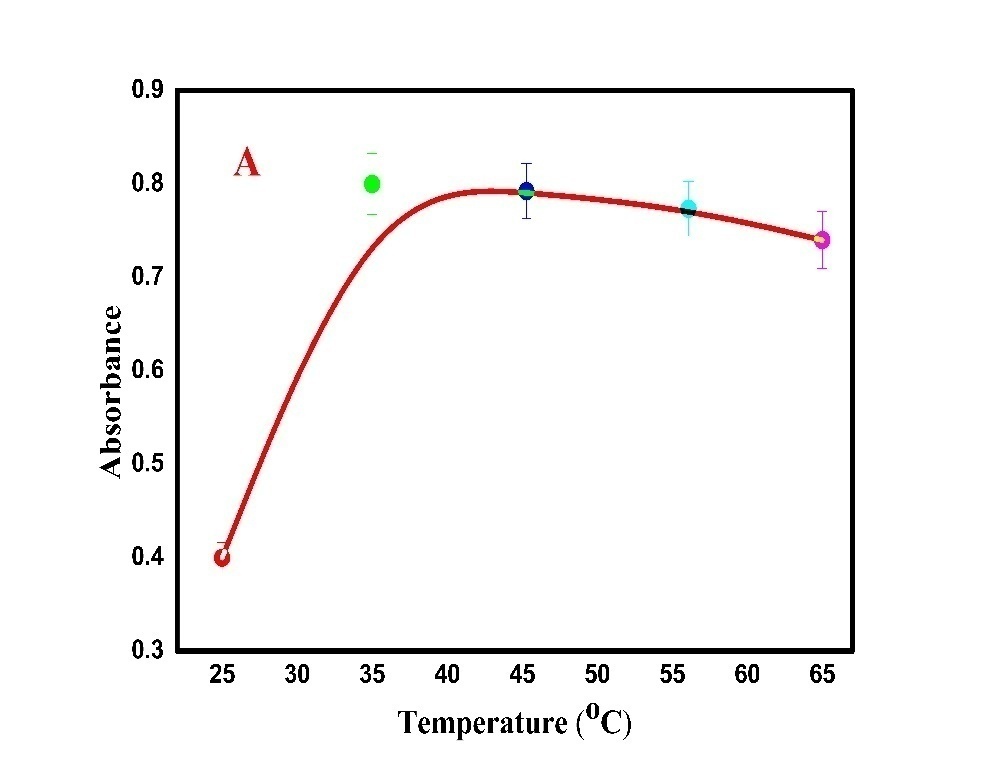 | | | | 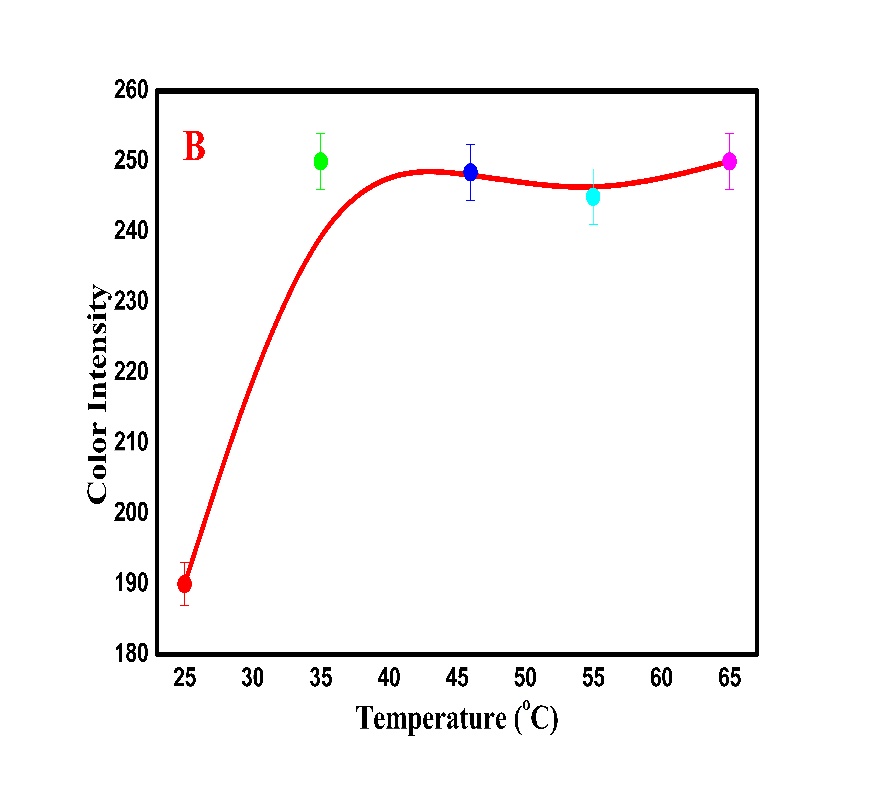  Fig. S4. |  |  |  |
|  | | | | |  |  |  |
| 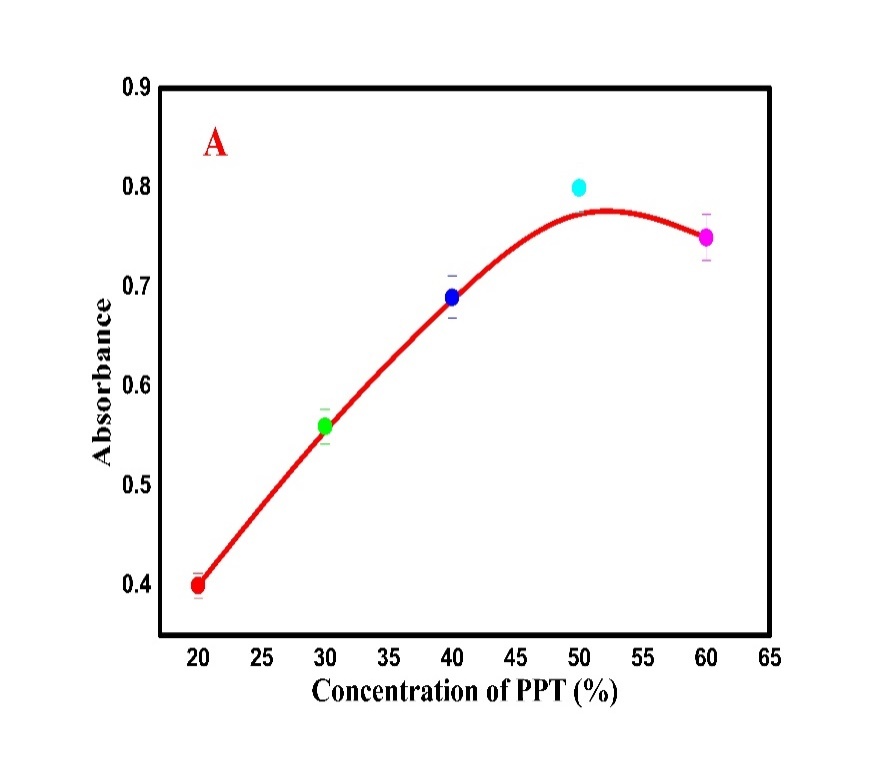 | | 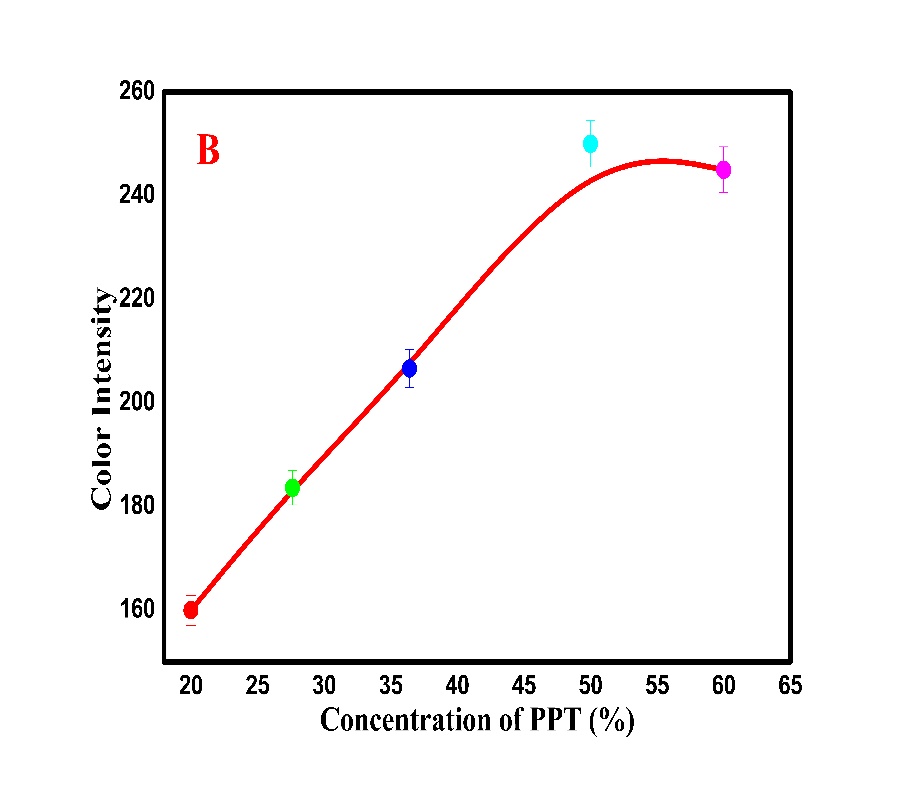  Fig. S5. | | |  |  |  |

| 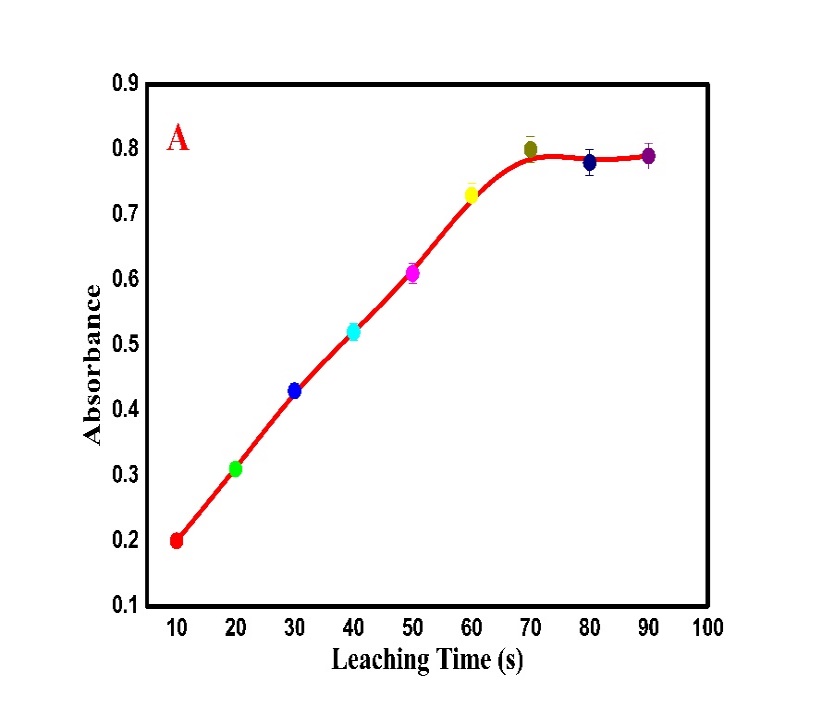 | 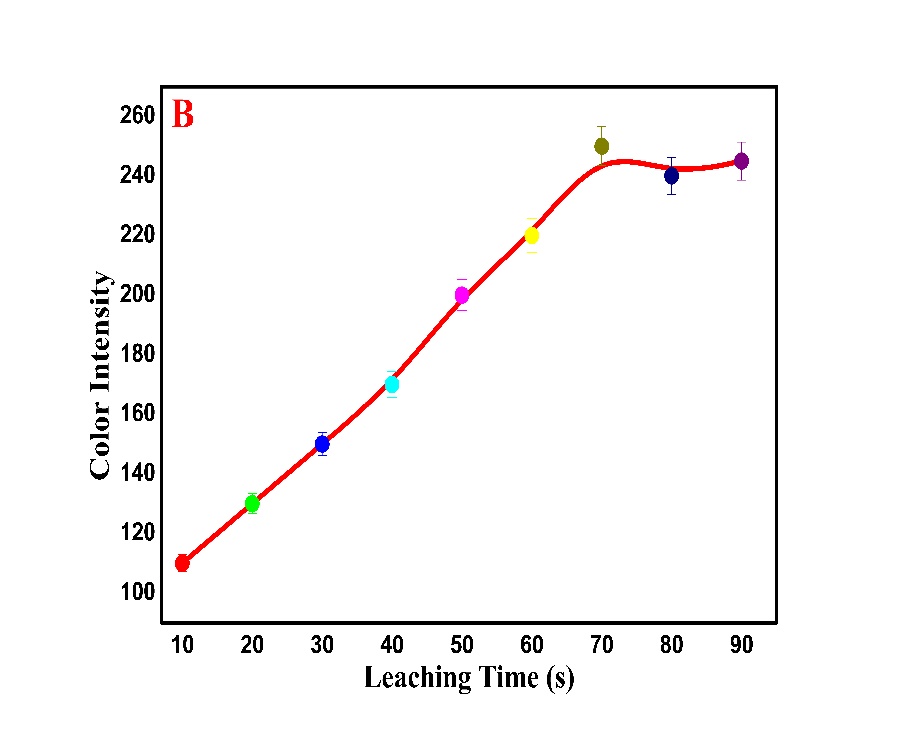 |
| --- | --- |
| Fig. S6.   | |

Fig. S7. Fig. S8.

**Tables**

**Table S1.**

| **Temp (k)** | **ΔG^0^ (KJ mol^−1^)** | **ΔH^0^ (KJ mol^−1^)** | **ΔS^0^ (KJ mol^−1^)** |
| --- | --- | --- | --- |
| **298.15** | - 5.39 | 29.12 | 0.113 |
| **308.15** | -6.02 |  |  |
| **318.15** | -6.64 |  |  |
| **328.15** | -7.04 |  |  |
| **338.15** | -7.51 |  |  |

**Table S2.**

|  | **Langmuir constants** | | | **Freundlich constants** | | |
| --- | --- | --- | --- | --- | --- | --- |
| **Optical**  **optode** | **KL** | **q_m_** | **R^2^** | **n** | **K_f_** | **R^2^** |
|  | **3080.8** | **73.2** | **0.997** | **1.9** | **2.12** | **0.89** |

**Table S3.**

| **Method and detection system** | **Linear Range**  **(μgL^-1^)** | **LOD**  **(μgL^-1^)** | **LOQ**  **(μgL^-1^)** | **RSD**  **(%)** | **Recovery (%)** | **Stability** | **Application** | **Ref** |
| --- | --- | --- | --- | --- | --- | --- | --- | --- |
| **CV-AAS** | 0.8-65 | 0.25 | 0.8 | 1.8 | _ | _ | Crude oil | 10 |
| **ICP-MS** | 0 -1.2 | 0.032 | _ | - | 99 | _ | Potable water | 11 |
| **ICP-AES** | 0.05- 1 | 10 | 20 | 2.25-7.4 | 109 | _ | Soil-Plant | 12 |
| **ICP-OES** | 0.005 -0.04 | 0.1 | 1 | 2-9 | 97 | _ | Fish | 13 |
| **Spectrophotometric** | 0.1-6 | 0.016 | 0.051 | 51 | _ | _ | Zamzam water and  Tap water | 14 |
| **CV- ICP-OES** | 0-10 | 0.02 | 0.07 | _ | 105 | _ | Waste water | 15 |
| **DIC-Histogram** | 0.005 -5000 | 0.066 | 0.22 | 1.31 | 98.1- 99.7 | 2 months | Fish, Soil and  Tap, Nile,  Sea water | This work |

**CV-AAS**; Cold Vapor Atomic Absorption Spectrometry, **ICP-MS**; inductively coupled plasma massspectrometry,**ICP-AES**; inductively coupled plasma atomic emission spectrometer, **ICP-OES**;inductively coupled plasma optical emission spectrometer, **DIC**; digital color analysis.

**Table S4.**

| Samples | Amount added  (μg L^-1^) | Found (μg L^-1^) ^a^ | | | | F.test | t.test |
| --- | --- | --- | --- | --- | --- | --- | --- |
|  |  | Optode technique | Recovery % | ICP-OES technique | Recovery % |  |  |
| River Nile water  Tap water  Seawater  Fish muscles  Cucumber  Soil (around river nile ) | 0  5.0  10.0  0  5.0  10.0  0  5.0  10.0  0  5.0  10.0  0  5.0  10.0  0  5.0  10,0 | 0.35  5.29  10.3  0.23  5.12  10.1  0.28  5.16  10.12  2.63  7.5  12.54  0.08  5.01  10.01  1.45  6.42  11,42 | -  98.8  99,5  _  97.8  98.7  _  97.7  97.27  _  98.2  99.2  _  98.6  99.3  _  99.5  99.7 | 0.31  5.28  10.15  0.2  5.21  10.16  0.27  5.20  10.1  2.58  7.45  12.41  0.08  4.98  10.06  1.43  6.40  11.41 | _  99.4  98.4  _  100.1  99.6  _  98.6  98.3  _  98.28  98.6  _  98.03  99.8  _  99.5  99.8 | _  1.8  2.1  _  2.05  1.55  _  2.03  _  1.65  1.88  _  1.74  1.8  _  2.02  204 | _  0.86  1.07  _  1.02  1.6  _  1.0  _  0.83  1.04  _  1.01  1.1  _  1.1  0.8 |

^a^ Average of four determination
